# Neurobiologische und neurochemische Mechanismen der Placeboanalgesie

**DOI:** 10.1007/s00482-022-00630-4

**Published:** 2022-03-17

**Authors:** Livia Asan, Ulrike Bingel, Angelika Kunkel

**Affiliations:** Klinik für Neurologie und Center for Translational Neuro- and Behavioral Sciences (C-TNBS), Unversitätsmedizin Essen, Hufelandstraße 55, 45147 Essen, Deutschland

**Keywords:** Schmerz, Nozizeption, Erwartung, Opioide, Dopamin, Pain, Nociception, Expectation, Opioids, Dopamine

## Abstract

**Hintergrund:**

Die Wirksamkeit von Schmerztherapien kann durch behandlungsbezogene Erwartungen wesentlich moduliert werden. Dies wird besonders unter Betrachtung des starken Placeboeffekts bei Schmerzen deutlich (die sog. Placeboanalgesie).

**Fragestellung:**

Was ist bisher über die neurobiologischen Grundlagen der Placeboanalgesie und die beteiligten neurochemischen Transmittersysteme bekannt?

**Material und Methoden:**

Fokussierte Auswahl wesentlicher Schlüsselpublikationen und strukturierte Darlegung mechanistischer Konzepte und aktueller Theorien basierend auf neuester Evidenz.

**Ergebnisse:**

In experimentellen Studien konnte die Wirkung der Placeboanalgesie insbesondere durch bildgebende Verfahren über eine Aktivitätsänderung in Hirnarealen der Schmerzverarbeitung und der kognitiven Kontrolle beschrieben werden. Beteiligte Neurotransmitter sind körpereigene Opioide und das Dopaminsystem.

**Schlussfolgerungen:**

Die Placeboanalgesie ist mit komplexen neurobiologischen und -physiologischen Mechanismen verbunden. Das Verständnis dieser Prozesse sollte gezielt genutzt werden, um therapeutische Ansätze in der Schmerzmedizin zu optimieren.

## Abstract (en)

**Background:**

The efficacy of pain therapies can be substantially modulated by treatment expectations, which is reflected by the substantial placebo effects observed in pain (so called placebo analgesia).

**Question:**

What is currently known about the neurobiological and neurochemical mechanisms underlying placebo analgesia?

**Materials and methods:**

A focused presentation of key publications in the field embedded in a structured overview of the mechanistic concepts and current theories according to recent evidence.

**Results:**

Experimental studies with functional neuroimaging showed that the effect of placebo analgesia is reflected by changes in brain activity related to pain processing and cognitive control. The important neurotransmitters involved include opioids and dopamine.

**Conclusion:**

Placebo analgesia is associated with complex neurobiological and -physiological mechanisms. An advanced comprehension of these processes should be applied to optimize existing and future therapeutic approaches in pain therapy.

In einer kürzlich durchgeführten klinisch-experimentellen Studie wurde PatientInnen mit chronischen Rückenschmerzen eine Infusion verabreicht, die als wirksames Schmerzmittel angekündigt wurde, aber tatsächlich keinen Wirkstoff beinhaltete; sie erhielten also ein Placebo [1]. Nach dieser Scheinmedikation kam es zu einer Reduktion ihrer Schmerzen von bis zu 54 %. Der Effekt der Placebobehandlung war damit vergleichbar mit der Analgesie durch Opioide, die zu den stärksten verfügbaren Schmerzmitteln zählen [2]. Wie kommen solche beeindruckenden Effekte zustande?

Der Begriff des Placebos wurde bei der Verwendung von Scheinbehandlungen in klinischen Studien geprägt. In einer traditionellen Sichtweise hängt der Erfolg einer medizinischen Behandlung nahezu ausschließlich von der jeweiligen Erkrankung und der spezifischen Wirkweise der Therapie, etwa den pharmakologischen Eigenschaften einer Medikation, ab. Ein „wahrer“ medizinischer Effekt einer Intervention bestehe demnach nur dann, wenn diese nachgewiesenermaßen besser wirke als ein Placebo.

Entgegen dieser Auffassung kann man allerdings häufig auch klinisch relevante Verbesserungen in dem Studienarm der Placebobehandlung beobachten. Dieser so genannte „Placeboeffekt“ kann gerade bei der Erprobung von Schmerzmedikamenten so stark sein, dass ein eigentlich potentes Analgetikum als scheinbar unwirksam aus Studien hervorgeht, weil es sich zu gering von der Placebowirkung unterscheidet. Da in den allermeisten Studien eine reine Beobachtungsgruppe ohne jegliche Intervention fehlt („natural history group“), kann nicht beziffert werden, zu welchem Anteil diese Effekte einer spontanen Symptombesserung (Spontanremission) im Rahmen der natürlichen Fluktuation der Krankheitsausprägung entspricht und zu welchem Anteil die Placebobehandlung zu einer Verbesserung führt [3]. Viel spricht aber dafür, dass bei Scheinbehandelten die Annahme, womöglich ein effektives Therapeutikum zu erhalten, einen großen Teil dieser Wirkung bedingt. So war auch in der oben dargestellten Studie der Placeboeffekt zur Besserung der chronischen Rückenschmerzen umso größer, je positiver die Erwartungen der PatientInnen bezüglich der Schmerzlinderung durch die Infusion war. Die therapiebezogene positive Erwartung ist also ein kritischer Mediator des Placeboeffekts.

## Mehr Sein als Schein – die Macht der Erwartung

**Figure 1:**
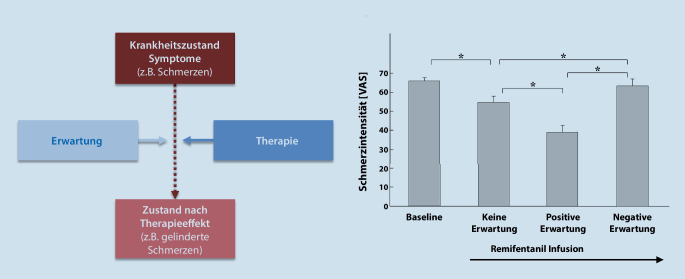

Tatsächlich ist der Placeboeffekt auch abseits von Studien in der täglichen klinischen Praxis bei erprobten Therapien und zur Routine gehörenden medizinischen Maßnahmen präsent. Innerhalb der letzten zwei Jahrzehnte konnten zahlreiche Untersuchungen belegen, dass die Erwartungshaltung gegenüber einer durchgeführten Behandlung ein maßgeblicher Modulator des Therapieerfolgs ist (Abb. 1a; [4, 5]). Der Schmerz gehört dabei zu den Systemen, die insgesamt besonders ausgeprägte Placeboantworten aufweisen. Eine Studie illustrierte dies beispielsweise dadurch, dass der analgetische Effekt des potenten Opioids Remifentanil wesentlich verstärkt werden konnte, wenn ProbandInnen aktiv über den Beginn der Infusion des starken Schmerzmittels informiert wurden (Abb. 1b; [6]). Sie nahmen einen Hitzereiz auf der Haut unter dieser bewusst erlebten Behandlung als weniger schmerzhaft wahr, als wenn die Verabreichung des Medikaments ohne deren Kenntnis ablief. Wurde hingegen die Infusion vermeintlich gestoppt, aber in Wirklichkeit weiter verabreicht, setzte die pharmakologische Wirkung von Remifentanil sogar nahezu vollständig aus. Die Hitzereize glichen dann in ihrer wahrgenommenen Intensität denen ohne jegliche Opioidbehandlung. Dieses Beispiel belegt, dass die therapiebezogene Erwartung als treibende Wirkvariable des Placeboeffekts durch verbale Instruktion wesentlich bestimmt werden kann. Darüber hinaus spielen positive Vorerfahrungen bezüglich einer Behandlung eine besondere Rolle. Dieser Aspekt kann u. a. durch die klassische Konditionierung erklärt werden: Der positiv konnotierte Reiz, hier die Einnahme der Tablette, induziert auch dann Linderung, wenn diese gar keinen aktiven Wirkstoff enthält. Die Effekte einer solchen „pharmakologischen Konditionierung“ wurden bereits 1999 untersucht [7]: ProbandInnen erhielten die Aufgabe, einen durch eine Blutdruckmanschette am Oberarm ausgelösten Ischämieschmerz am Arm möglichst lange auszuhalten. Ein Placebo erhöhte die Schmerztoleranz besonders dann, wenn in vorherigen Testdurchläufen ein positiver Effekt von Morphinen oder von NSAR (nichtsteroidalen Antirheumatika) erlebt wurde. In einer klinischen Studie konnte die Dosis an benötigten Morphinäquivalenten zur Schmerzkontrolle nach Polytrauma oder Rückenmarksverletzung durch das zusätzliche Angebot von Placebos drei Tage nach ausschließlicher Oxycodontherapie signifikant verringert werden [8]. Bemerkenswert hierbei: Die PatientInnen wussten, dass es sich dann um eine wirkstofflose Kapsel handelte. Die StudienleiterInnen nutzten für die Konditionierung gezielte Kontextreize aus: Die Oxycodontabletten waren von Anfang an mit dem Geruch von Kardamonöl gepaart, so auch die folgenden Kapseln der Placebobehandlung. Der Geruch unterstützte somit als konditionierter Stimulus, der mit Schmerzlinderung assoziiert wurde, die Placeboanalgesie.

Neben Lernen- und Konditionierungsprozessen spielen noch weitere Kontextfaktoren in die Konstruktion einer Erwartung mit hinein: etwa das Therapieumfeld mit professionellem Untersuchungszimmer, die Dauer der ärztlichen Zuwendung, eine empathische Kommunikation oder auch die äußere Gestaltung einer Medikamentenpackung. Insgesamt geht man davon aus, dass es sich bei dem Placeboeffekt um das Ergebnis eines Zusammenspiels verschiedener psychologischer Determinanten handelt, die sich in ihrer Gesamtheit über die Erwartung ausdrücken lassen. Der hier verwendete Erwartungsbegriff kann dabei sowohl bewusste als auch unbewusste Komponenten beinhalten.

Die therapeutische Nutzung von Placeboeffekten für die Schmerztherapie könnte großes Potenzial für die Versorgung von PatientInnen mit akuten oder chronischen Schmerzen bergen. Dafür sind genaue Kenntnisse der Mechanismen der Placeboanalgesie unverzichtbar. In diesem Artikel möchten wir einen Überblick über den bisherigen Wissensstand zu neurobiologischen und neurochemischen Grundlagen der Placeboanalgesie liefern. Ein Anspruch auf Vollständigkeit besteht hierbei nicht; vielmehr sollen wichtige grundlegende Prinzipien beleuchtet werden, um die Placeboanalgesie mechanistisch greifbar zu machen.

Das Schmerzsystem ist aufgrund der nachgewiesenen Auslösbarkeit von Placeboeffekten, der breiten klinischen Relevanz und der experimentellen Zugänglichkeit in der Placeboforschung bisher das am besten untersuchte System. Psychoneurobiologische Grundlagen der Placeboanalgesie konnten insbesondere in Studien mit gesunden ProbandInnen, denen gezielt Schmerzreize appliziert wurden, bereits gut charakterisiert werden.

Besonders naheliegend ist zunächst die Frage, ob sich die reduzierte Wahrnehmung von Schmerzen durch Anwendung einer Placebobehandlung überhaupt im zentralen Nervensystem widerspiegelt. Ändert sich tatsächlich die Aktivität schmerzverarbeitender Netzwerke im Gehirn? Welche Hirnareale vermitteln eine erwartungsinduzierte Modulation der Wahrnehmung? Und gibt es konkrete neurochemische Prozesse, die an der Wirkung zentral beteiligt sind? Ein tieferes Verständnis über die Wirkung von Placebobehandlungen soll dabei helfen, die „Echtheit“ und Relevanz ihres Effekts anzuerkennen und ihn bestmöglich therapeutisch zum Vorteil unserer PatientInnen zu nutzen.

## Ein Placebo hemmt die sensorische Schmerzverarbeitung

**Figure 2:**
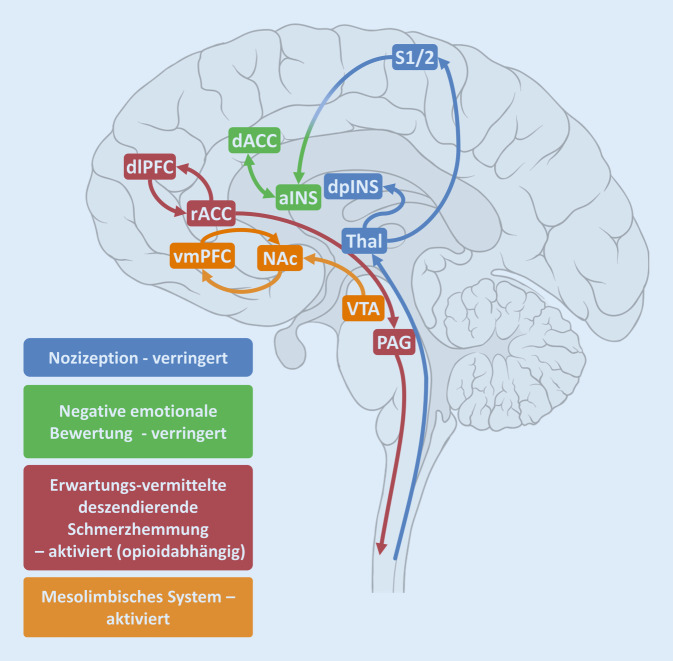

Viele Fragen konnten in den letzten zwei Jahrzehnten mithilfe funktioneller Hirnbildgebung beantwortet werden. In der funktionellen Magnetresonanztomografie (fMRT) kann nichtinvasiv, über die Änderungen der magnetischen Eigenschaften von Blutbestandteilen durch lokalen Sauerstoffverbrauch, indirekt die neuronale Aktivität verschiedener Hirnareale gemessen werden. Durch den Vergleich der Aktivierungen während der Applikation von schmerzhaften Reizen mit (Placebobedingung) und ohne (Kontrollbedingung) Anwendung einer Placebobehandlung lassen sich placebospezifische Unterschiede herausstellen. In einem oft genutzten experimentellen Paradigma werden beispielsweise schmerzhafte Hitzereize auf unterschiedliche Stellen der Haut des Unterarms appliziert [9–12]. Auf einer dieser Stellen wird zuvor, nach Ankündigung, eine Salbe mit vermeintlich analgetischen Inhaltsstoffen aufgetragen (Placebobedingung), auf der anderen eine ebenso inerte Kontrollsalbe, der keine aktiven Substanzen zugesprochen werden (Kontrollbedingung), wobei die ProbandInnen die verspürte Schmerzintensität nach jedem Reiz bewerten. Obwohl sich tatsächlich beide Salben gleichen und keine von ihnen einen Wirkstoff beinhaltet, wird der Schmerz in der Placebobedingung im Mittel als geringer empfunden. Bei diesen und ähnlichen Placeboparadigmen kann nun gleichzeitig die Hirnaktivität im MRT-Scanner während des Experiments untersucht werden. Durch solche Versuche konnte gezeigt werden, dass Hirnareale, die in der primären Verarbeitung des eingehenden nozizeptiven Signals für die sensible und diskriminative Komponenten der Schmerzwahrnehmung involviert sind (Thalamus, S2, dorsale posteriore Insula; siehe Abb. 2), tatsächlich eine geringere Aktivierung nach Schmerzreizen unter Placebo im Vergleich zur Kontrollkondition aufweisen [12]. Wir können also davon ausgehen, dass die durch positive Erwartung induzierte Schmerzlinderung während einer Placebobehandlung tatsächlich – zumindest partiell – auf einer veränderten Verarbeitung nozizeptiver Reize beruht.

Wie genau diese Modulation bewerkstelligt wird und durch welche Hirnareale und neurochemischen Prozesse sie primär vermittelt wird, bleibt bei dieser Betrachtung zunächst offen.

## Das absteigende schmerzhemmende System

**Figure 3:**
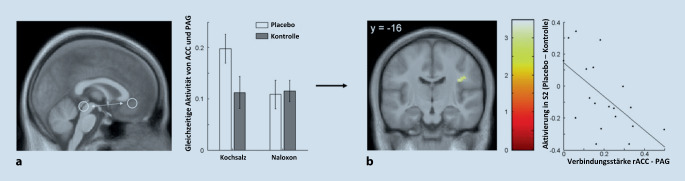

Von Hirnarealen, die für die Hemmung bzw. Modulation der Schmerzverarbeitung zuständig sind, erwartet man mehr Aktivität, je stärker der Schmerz während der Placeboanalgesie gelindert ist. In der Tat wurden in zahlreichen fMRT-Studien auch genau solche Regionen identifiziert. Eine Schlüsselfunktion für die körpereigene Schmerzmodulation inklusive der Placeboanalgesie wird dem dorsolateralen präfrontalen Cortex (dlPFC) zugeschrieben, welcher u. a. für die innere Repräsentation von Zielen und Erwartungen zuständig ist und rege mit anderen Hirnarealen kommuniziert. Hier zeigte sich in der Placebobedingung eine höhere Aktivierung während der Antizipationsphase von Schmerzen, also nach Ankündigung und kurz vor Applikation eines Schmerzreizes [12]. Je größer die Aktivierung war, desto effektiver entpuppte sich zudem die Schmerzlinderung durch die Placebobehandlung. Der dlPFC kodierte hier also vermutlich Aspekte der positiven Erwartungshaltung. *Während* eines Schmerzreizes wiederum wurde der rostrale anteriore cinguläre Cortex (rACC) vermehrt rekrutiert (Abb. 3a; [10]). Er scheint also die neurale Basis für die endogene Schmerzkontrolle durch Kognition darzustellen [13]. Über Verbindungen zum Hirnstamm und von dort absteigende Bahnen wurde einer weiteren Studie zufolge die synaptische Weiterleitung der aus der Peripherie einkommenden nozizeptiven Signale direkt auf Rückenmarkssegmentebene gehemmt [14]. Dieses sog. deszendierende schmerzhemmende System stellt eine „Top-Down“-Regulation dar, durch die eine Modulation bereits auf der untersten Ebene der zentralen Verarbeitung stattfindet. Die unmittelbare Folge hiervon ist die oben beschriebene placeboassoziierte verminderte Aktivierung von Hirnzentren der Nozizeption nach Schmerzreiz (Abb. 3b).

## Opioide sind wichtige Mediatoren der Placeboanalgesie

Es war bereits früh bekannt, dass das körpereigene Opioidsystem eine besondere Rolle in der Vermittlung der Placeboanalgesie spielt. Levine konnte 1978 zeigen, dass die durch ein Placebo erreichte Analgesie nach operativer Zahnextraktion mit der Gabe des Opioidantagonisten Naloxon verringert werden konnte [15]. Dies war der erste Hinweis darauf, dass endogene Opioide den Placeboeffekt vermitteln könnten, und Folgeuntersuchungen konnten diese Beobachtungen replizieren [16]. Um den Einfluss von Neuromodulatoren und Neurotransmittern genauer zu untersuchen, sind Methoden der sog. „molekularen Bildgebung“ wegweisend. Hierzu gehört die Positronenemissionstomografie (PET), die die Verteilung von verabreichten radioaktiven Substanzen und damit die lokale Belegung bestimmter Rezeptoren im Gehirn sichtbar machen kann. In einer Studie wurden durch eine Injektion hypertoner Kochsalzlösung Schmerzen im Kaumuskel ausgelöst. Mit der PET wurde die Verteilung des verabreichten radioaktiv markierten Carfentanil im Gehirn dargestellt, welches mit den körpereigenen Opioiden um die Bindungsstellen der µ‑Opioid-Rezeptoren konkurriert. Während der Infusion eines Placeboanalgetikums und der Suggestion einer dadurch erreichten Schmerzlinderung wurde eine Opioidaktivierung im dlPFC und dem rACC während der Infusion eines Placeboanalgetikums beobachtet [17]. Diese Aktivierung vermittelte dann über Verbindungen zum periaquäduktalen Grau im Hirnstamm wiederum eine Opioidausschüttung und initiierte damit die absteigende Schmerzhemmung (Abb. 2). Endogene Opioide konnten also als der neurochemische Generator der oben beschriebenen, durch positive Erwartung ausgelösten Top-Down-Regulation identifiziert werden. Interessanterweise waren diese Prozesse nahezu identisch mit denen, die nach der direkten Gabe von opioidhaltigen Medikamenten sichtbar wurden [18]. Die Erwartung einer Schmerzlinderung wirkt also in der Endstrecke über die Ansteuerung derselben Mechanismen, die auch den potenten analgetischen Effekt von Opioiden vermitteln. Bestätigt wurde dieser Zusammenhang durch die Beobachtung, dass sich die Reduktion der Placeboantwort durch die Gabe von Naloxon auch in einer Aktivitätsverringerung entlang dieser neuralen Bahnen in der funktionellen Bildgebung widerspiegelte (Abb. 3a; [10]).

## Nicht die ganze Wahrheit

Gänzlich aufheben konnte Naloxon die Placeboanalgesie allerdings nicht. Zudem korrelierte die Minderaktivierung der nozizeptiven Hirnareale tatsächlich nur gering mit dem Ausmaß der empfundenen Schmerzlinderung auf individueller Ebene [19]. Es liegt daher nahe, dass die Modulation der nozizeptiven Signaltransmission nicht die einzige Erklärung sein kann [20].

Wir wissen, dass wir Schmerz nicht nur als physischen Reiz einer bestimmten Intensität an einer Körperstelle wahrnehmen. Vielmehr wirkt er sich auch auf das emotionale Erleben, die Aufmerksamkeit und weitere kognitive Leistungen aus. Diese komplexeren Dimensionen des Schmerzerlebens werden durch die Interaktion von distinkten, höheren assoziativen Hirnregionen repräsentiert, die keineswegs spezifisch für Schmerzen sind. Dazu gehören der dorsale anteriore cinguläre Cortex (dACC) und die anteriore Insula. Tatsächlich führt eine Placebobehandlung auch hier zu einer reduzierten Aktivierung im Vergleich zur Kontrolle (Abb. 2; [10, 12, 13]). Es wird vermutet, dass dieser Effekt durch Integration von Informationen aus verschiedenen „niedrig-“ bis „hochkomplexen“ Verarbeitungsstufen von Nozizeption, Kognition, Emotion und, nicht zuletzt, der subjektiven Bewertung zustande kommt. An dieser Stelle wird nun zunehmend auch subkortikalen Strukturen, wie den Basalganglien, der Amygdala und weiteren Teilen des limbischen Systems, eine Bedeutung beigemessen (für eine Übersicht siehe [21]). In diesem Zusammenhang nehmen aktuell das Dopaminsystem und damit assoziierte Kerngebiete im Gehirn eine besondere Stellung ein.

## Dopamin: Placeboanalgesie als erstrebenswerte Belohnung?

**Figure 4:**
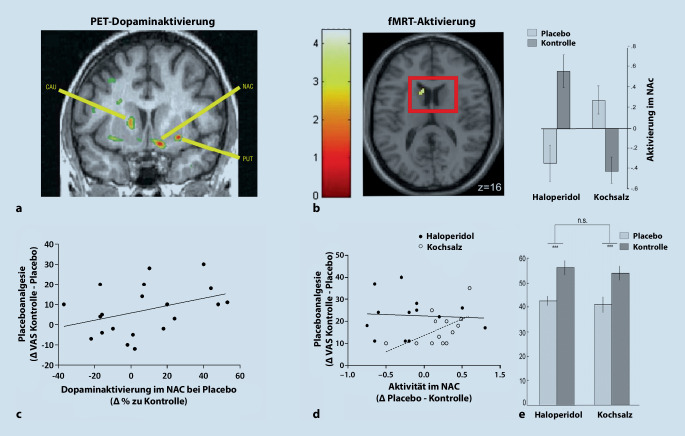

Die erste Studie, die die Rolle von Dopamin für die Placeboanalgesie untersuchte, zeigte mittels PET, dass die Infusion eines vorgeblichen Anästhetikums vor einem angekündigten Schmerzreiz mit der Aktivierung von D2-/D3-Rezeptoren im ventralen Striatum, im Speziellen dem Bereich des Nucleus accumbens (NAc), einhergeht [22]. In dieser Phase der Antizipation von Schmerz korrelierte die Stärke der Dopamintransmission sowohl mit der individuellen Erwartung der ProbandInnen bezüglich des schmerzlindernden Effekts als auch mit der letztlich wahrgenommenen Placeboanalgesie positiv (Abb. 4a).

Der NAc liegt im ventralen Striatum (VS) und ist Teil des sog. mesolimbischen Systems. Er erhält dopaminerge Projektionen aus dem Mittelhirn und unterhält Verbindungen zu limbischen Arealen wie dem cingulären Cortex. Dem mesolimbischen System wird eine bedeutsame Rolle im Belohnungssystem zugeschrieben. Folgt eine Belohnung auf einen neutralen Stimulus, sorgt die Dopamintransmission im mesolimbischen System, so vermutet man, für die Umdeutung des zuvor neutralen Reizes zu einem attraktiven Reiz. Die Wahrnehmung dieses Stimulus löse dann eine hohe Motivation zum Erreichen der Belohnung aus: Die sog. „Anreiz-Salienz“ des Stimulus scheint erhöht zu werden [23]. Dopamin beeinflusst demnach also vor allem die Ausbildung des *Wollens* einer Belohnung (Belohnungsmotivation, *reward wanting*), die durch einen Stimulus angezeigt wird [24]. Wenn die Dopaminwirkung im NAc beispielsweise pharmakologisch durch Sulpirid gestört wird, reagieren ProbandInnen weniger schnell und weniger präzise auf einen Stimulus, der mit einem Geldgewinn assoziiert wird, als die Kontrollgruppe ohne Sulpirid [25]. Sie hatten die Bedeutung der Stimuli zwar genauso gut gelernt, aber waren im Schnitt unmotivierter, auf den Stimulus einzugehen. Während des belohnungsassoziierten Lernens scheint die Ausschüttung von Dopamin im NAc zunächst dann hochzuschießen, wenn nach einem Stimulus *unerwarteterweise* gerade eine Belohnung eingetreten ist. Ist diese Assoziation einmal verinnerlicht, würde Dopamin dann bereits während der Erwartung der Belohnung ausgeschüttet werden, sobald der Stimulus erneut gesichtet wird [26, 27].

Die Beobachtung, dass die Aktivierung des mesolimbischen Systems auch das Ansprechen auf eine Placebobehandlung vorhersagen konnte, spricht für eine Relevanz dieses Systems für das Empfinden der Placeboanalgesie [28]. Es werden anscheinend die neuralen Pfade der Belohnungsmotivation genutzt. Dieser Zusammenhang konnte durch weitere interessante Beobachtungen illustriert werden: Die Placeboanalgesie eines Probanden oder einer Probandin konnte allein durch das Ausmaß der Aktivierung des NAc während der Erwartung eines Geldgewinns in einem anderen Versuch vorhergesagt werden [22]. Auch Persönlichkeitseigenschaften, die eng mit der Funktion von Dopamin verknüpft sind, wie etwa Neugierde, Antrieb und Empfänglichkeit für Belohnungen, scheinen psychologische Determinanten der Placeboanalgesie zu sein. Sogar die Anatomie des mesolimbischen Systems ist mit der Placeboantwort bei Schmerz assoziiert: Je größer der NAc ist, desto wirksamer ist eine Placebobehandlung [29]. Die bisherigen Evidenzen lassen folgende Theorie zu: Ein Placebo könnte einen Stimulus darstellen, dessen positive Signalfunktion für eine Schmerzlinderung (=die Belohnung) durch Suggestion oder durch eigene gute Vorerfahrung gelernt wird. Die Assoziation zwischen Placebo und Analgesie wird dabei dopaminabhängig verstärkt und gewinnt vor allem an motivationaler Bedeutung. Das Placebo würde hiernach zu einem appetitiven Reiz werden, dessen angezeigte Belohnung in Form von verringerten Schmerzen erreicht werden *will, *und dies hat eine erfolgreichere Placeboanalgesie zur Folge.

Um die kausale Relevanz von Dopamin zu prüfen, eignen sich Placeboexperimente mit pharmakologischen Interventionen, die in die Wirkung oder die Verfügbarkeit des Neurotransmitters eingreifen. Zunächst gilt es herauszufinden, ob sich der analgetische Effekt der Placebobehandlung direkt durch Hemmung der Dopaminübertragung unterdrücken lässt. In einer Studie wurde durch die Verabreichung von Haloperidol, welches die Dopaminaktivität im Bereich des NAc nachweisbar reduzierte, genau dies untersucht [11]. Allerdings war eine Placebosalbe gegen Hitzeschmerz hier analgetisch trotzdem genauso effektiv wie in der Kontrollgruppe ohne Haloperidol (Abb. 4b, c). Die Dopaminausschüttung *während* einer Placebobehandlung scheint also *nicht notwendig* für den analgetischen Effekt zu sein. Ähnlich konnte in einem Versuch mit PatientInnen, die unter chronischen neuropathischen Schmerzen leiden, durch die Gabe von Haloperidol oder L‑Dopa (eine Vorstufe von Dopamin) keine abschwächende oder verstärkende Auswirkung auf die Placeboanalgesie festgestellt werden [30]. Es ist allerdings denkbar, dass sich, in Analogie zu dessen Rolle im belohnungsbezogenen Lernen, die eigentlich relevante Beteiligung von Dopamin früher ereignet, nämlich schon beim *Erlernen* des Stimulus-Ergebnis-Zusammenhangs. Demnach könnte es also an der Bildung der placeboassoziierten positiven Erwartung beteiligt sein. Es sind weitere Studien nötig, um die Mechanismen einer Regulation durch Dopamin genauer zu charakterisieren.

## Die Chemie stimmt

Während Dopamin, wie beschrieben, vermutlich über eine intrinsische motivationale Komponente wirkt, könnte eine andere Substanz eine Stellschraube für das Ausmaß an Vertrauen und emotionaler Bindung gegenüber Behandelnden darstellen: Oxytocin. Bekannt als das „Kuschelhormon“ konnte Oxytocin nach Applikation durch ein Nasenspray die analgetische Wirkung einer Placebosalbe, die zuvor durch einen ärztlichen Versuchsleiter eingeführt wurde, verbessern [31].

Cannabinoide scheinen verschiedene Aspekte der Placeboanalgesie zu vermitteln. Rimonabant, ein CB1-Cannabinoid-Rezeptor-Blocker, konnte diejenige Placeboanalgesie blocken, die nach pharmakologischer Konditionierung mit dem nichtsteroidalen Antirheumatikum (NSAR) Ketorolac ausgelöst worden war [32]. Auf die Placeboanalgesie nach Konditionierung mit einem Opioid hatte Rimonabant hingegen keinen Einfluss. Daraus lässt sich ableiten, dass endogene Cannabinoide den analgetischen Effekt bei Placebogabe nach analgetischen Vorerfahrungen mit NSAR aufrechterhalten. Darüber hinaus üben Cannabinoide einer Studie zufolge womöglich auch bei Vorliegen von positiv konnotierten Schmerzen einen günstigen Einfluss auf die Schmerzbewertung aus. Wird in der eigenen Vorstellung der Erfolg eines Krafttrainings dann erhöht, wenn die Muskeln richtig schmerzen, erträgt man diese fast gerne [33]; die durch diese optimistische Sichtweise erhöhte Schmerztoleranz konnte ebenfalls durch Rimonabant geschwächt werden.

Es zeigt sich, dass verschiedene Aspekte der Placeboanalgesie durch unterschiedliche neurochemische Modulatoren vermittelt werden können. Je nach Situation, nach Modalität der Placeboanwendung und nach persönlichen und individuellen Faktoren beeinflusst ein wohl abgestimmter „Cocktail“ dieser Botenstoffe, unter Ansteuerung verschiedener neuraler Netzwerke, die Schmerzwahrnehmung über die positive Therapieerwartung.

## Ausblick und offene Fragen

Die Kenntnis der genauen Einflussnahme von Neurotransmittern auf die Ausbildung und Stärke von Placeboantworten birgt die Hoffnung, diese durch gezielte, individualisierte Pharmakotherapie zu therapeutischem Nutzen maximieren zu können. Ansätze für einen „Boost“ der Placebokomponente mit einer zusätzlich zu einem Analgetikum begleitenden Gabe von Medikamenten, die beispielsweise auf den Dopamin- oder Oxytocinhaushalt wirken, müssen dafür weiter intensiv beforscht werden. Ein weiteres wichtiges Entwicklungsfeld ist die Identifikation von Placebomechanismen bei klinischen Populationen, zu denen bisher nur sehr vereinzelt Daten vorliegen. Zur Verwirklichung einer individualisierten, gezielten Therapie von PatientInnen ist es außerdem wichtig, Prädiktoren zu identifizieren, die das individuelle Ansprechen auf Placeboanwendungen vorhersagen können. Diese Notwendigkeit wird angesichts der sehr interindividuell sehr variablen Kapazität für Placeboanalgesie mit deutlich profitierenden Respondern, aber auch Non-Respondern ersichtlich. Eine niedrigere Dosis an Analgetika unter Anwendung endogener Schmerzkontrolle ist beispielsweise bei potenziellen Respondern sinnvoll, während Non-Responder von dieser Option nicht profitieren, wie das beispielsweise bei Alzheimer-Patienten der Fall zu sein scheint [34]. Es gibt Hinweise, dass gewisse Persönlichkeitseigenschaften und psychische Zustände wie Nervosität, Stress, negative Emotionen den Einfluss von Erwartung auf Schmerzen beeinflussen können. Neben genetischen Faktoren könnten auch strukturelle und funktionelle neurobiologische Unterschiede für eine gute oder schlechte Placebokapazität veranlagen; hierzu zeigen Untersuchungen der Konnektivität zwischen Hirnarealen und der Integrität von Faserverbindungen erste spannende Ergebnisse, die aber noch weiter bestätigt und ergänzt werden müssen [35].

Durch die erläuterten Vorgehensweisen wie verbale Suggestion, empathische Zuwendung, positive Formulierungen und die Bildung eines professionellen therapeutischen Rahmens können Behandelnde aber bereits jetzt über eine positive Erwartungsbildung einen Einfluss auf den Therapieerfolg nehmen. Die Darstellung einiger relevanter neurobiologischer und neurochemischer Prozesse in diesem Artikel soll ein tieferes Verständnis für die objektivierbaren Mechanismen und Effekte sowie deren therapeutische *Relevanz* vermitteln und dazu ermutigen, diese nützlichen Aspekte als festen Bestandteil in der Schmerztherapie von PatientInnen aktiv zu nutzen.

## Fazit für die Praxis

- Placeboeffekte können akute und chronische Schmerzen bedeutsam verringern.
- Die wesentliche Wirkvariable der Placeboanalgesie ist die positive therapiebezogene Erwartung.
- Sie geht mit messbaren Veränderungen der Schmerzverarbeitung und -modulation im zentralen Nervensystem einher.
- Eine besondere Rolle spielen das Opioid- und das Dopaminsystem.
- Ein wichtiges Zukunftsfeld der Erforschung von Placebo- bzw. Erwartungseffekten ist das Verständnis von interindividuellen Unterschieden und Prädiktoren für die individuelle Placeboantwort.
